# Digital innovations for retinal care in diabetic retinopathy

**DOI:** 10.1007/s00592-022-01941-9

**Published:** 2022-08-12

**Authors:** Stela Vujosevic, Celeste Limoli, Livio Luzi, Paolo Nucci

**Affiliations:** 1 Department of Biomedical, Surgical and Dental Sciences, University of Milan, Milan, Italy; 2 Eye Clinic, IRCCS MultiMedica, Via San Vittore 12, 20123 Milan, Italy; 3 University of Milan, Milan, Italy; 4 Department of Biomedical Sciences for Health, University of Milan, Milan, Italy; 5 Department of Endocrinology, Nutrition and Metabolic Diseases, IRCCS MultiMedica, Milan, Italy

**Keywords:** Telemedicine, Teleophthalmology, Artificial intelligence, Diabetes mellitus, Diabetic retinopathy, Digital health

## Abstract

**Aim:**

The purpose of this review is to examine the applications of novel digital technology domains for the screening and management of patients with diabetic retinopathy (DR).

**Methods:**

A PubMed engine search was performed, using the terms “Telemedicine”, “Digital health”, “Telehealth”, “Telescreening”, “Artificial intelligence”, “Deep learning”, “Smartphone”, “Triage”, “Screening”, “Home-based”, “Monitoring”, “Ophthalmology”, “Diabetes”, “Diabetic Retinopathy”, “Retinal imaging”. Full-text English language studies from January 1, 2010, to February 1, 2022, and reference lists were considered for the conceptual framework of this review.

**Results:**

Diabetes mellitus and its eye complications, including DR, are particularly well suited to digital technologies, providing an ideal model for telehealth initiatives and real-world applications. The current development in the adoption of telemedicine, artificial intelligence and remote monitoring as an alternative to or in addition to traditional forms of care will be discussed.

**Conclusions:**

Advances in digital health have created an ecosystem ripe for telemedicine in the field of DR to thrive. Stakeholders and policymakers should adopt a participatory approach to ensure sustained implementation of these technologies after the COVID-19 pandemic. This article belongs to the Topical Collection "Diabetic Eye Disease", managed by Giuseppe Querques.

## The burden of diabetes mellitus and diabetic retinopathy

Diabetic retinopathy (DR) is the most common ocular complication caused by diabetes mellitus (DM) and a leading cause of preventable blindness globally [1]. Nearly 30% of DM patients suffer from DR with an expected increase in global DR rates of 55.6% from 2020 to 2045 [1].

Early detection through active screening and timely treatment of vision-threatening DR are the best ways to reduce the risk of vision loss [2]. The key strategy to detect DR is annual fundus examination, which can be performed through several methods and by different screeners as seen across countries worldwide [3, 4].

Great emphasis has been placed on telemedicine as a DR screening program to enable early detection and treatment of sight-threatening DR. DM and DR are particularly well suited to digital technologies, providing an ideal model for telehealth initiatives with real-world application [3]. The COVID-19 pandemic has led to an unprecedented adoption of telemedicine and telehealth, which would not have otherwise happened in such a short span of time [4].

The purpose of this review is to report on the current level of development in the adoption of telemedicine for the management of patients with DR, with a particular emphasis on digital advancements in screening methods. The best applications of telehealth models of care and the use of artificial intelligence (AI) for DR as an alternative to or in addition to traditional forms of care will be discussed.

## Search strategy and selection criteria

To identify potentially relevant published research in PubMed were searched from January 1, 2010, to February 1, 2022, with the following search terms: (“Telemedicine” [All fields]) OR (“Digital health” [All fields]) OR (“Telehealth” [All fields]) OR (“Telescreening” [All fields]) OR (“Artificial intelligence” [All fields]) OR (“Deep learning” [All fields]) OR (“Smartphone” [All fields]) AND (“Triage” [All fields]) OR (“Screening” [All fields]) OR (“Home-based” [All fields]) OR (“Monitoring” [All fields]) AND (“Ophthalmology” [All fields]) OR (“Diabetes” [All fields]) OR (“Diabetic Retinopathy” OR (“Retinal imaging” [All fields]) AND (English [Language]). Full-text English language studies and reference lists were considered for the conceptual framework of this review.

## Telemedicine in DR screening

Despite the recommendation for annual DR examination in patients with DM as a cost-effective method to reduce preventable blindness, screening is poorly implemented and is not likely to deal with the increasing demand in the future due to the escalating population with newly diagnosed DM [1]. Against this backdrop, the maturation of digital technologies, including AI approaches, and the further consolidation of telehealth have created an unprecedented ecosystem for new opportunities in screening, diagnosis, and management of DR [3, 4]. Therefore, new digital models of care have been quickly deployed to expand standards of eye care delivery for patients with DM by integrating existing methods to address the growing challenges of patient non-compliance and limited access to care.

Several screening programs worldwide have been successfully implemented using 1, 2, or 3 fundus fields (mostly 45°) for DR [5–7].

Ocular telehealth programs for DR rely on the acquisition of retinal images to determine the presence and severity of DR and diabetic macular edema (DME), mainly based on a store-and-forward approach [5].

The gold standard procedure to detect and study DR in clinical trials had been in the past the seven standard field stereoscopic Early Treatment Diabetic Retinopathy Study (ETDRS) mydriatic 35 mm color slides, and currently are digital fundus 30° color photographs (7F-ETDRS).

7F-ETDRS has proven to be cumbersome and time-consuming in the screening and clinical setting, and therefore, different imaging methods with distinct acquisition protocols have been explored for DR screening. Most screening services have established a two-field 45° digital color fundus photograph (CFP) strategy, showing a good balance between diagnostic accuracy, technical feasibility, and patient compliance. The non-mydriatic two-field approach is considered a practical strategy in DR screening programs using digital retinal imaging, with pupil dilation only when images are ungradable. Compared to the ETDRS gold standard, the sensitivity and specificity of two-field CFP were 96% and 89%, inferior to those of three-field CFP (92% and 96%), but superior to those of single-field CFP (78% and 86%) [6, 7].

The most accomplished nationwide DR screening program based on telemedicine has been established in the UK, with an uptake of above 80% of DM population [8]. After introducing this effective screening program, DR/maculopathy does not represent the major cause of certifiable blindness anymore in England and Wales among working-age adults, for the first time in the last 50 years [9].

Even if conventional 45° CFP is the most common approach to retinal image acquisition for DR screening, it has the shortcoming of providing limited visualization of the peripheral retina. Advances in non-mydriatic imaging have allowed overcoming this limit by means of ultra-wide-field (UWF) retinal imaging [10, 11]. UWF fundus photography on scanning laser ophthalmoscope (SLO) has been compared favorably with 7F-ETDRS photographs and has allowed identification of DR lesions outside the standard ETDRS fields, allowing the visualization of significantly greater area of the retina up to 200° coverage [12].

In addition, only one image is needed by means of UWF fundus camera and can be captured without pupil dilation. Therefore, non-mydriatic UWF imaging showed to be useful for DR ocular telehealth program by reducing the number of ungradable images, increasing the identification of DR nearly twofold, and identifying peripheral lesions suggesting more severe DR [13].

Teleophthalmology initiatives showed that including OCT can improve the efficiency of DR/DME screening programs. Given the advantages of the OCT device for DME assessment, OCT will be likely incorporated in well-established DR screening programs with screen-positive maculopathy [14, 15].

However, all the above-mentioned retinal imaging devices have limited use worldwide due to the high cost, limited portability, and lack of technical expertise. Novel hybrid telemedicine systems have been explored over the current design methods to allow a wider coverage for DR screening, such as diagnostic sets hard-mounted on vans. A combination of a mobile UWF cameras mounted on vehicles and fixed-location classic cameras in clinics has optimized the telemedicine health network and the use of current diagnostic resources [16].

Mobile diagnostic sets have been harnessed in China by the large-scale nationwide telemedicine-enabled screening program, including several primary sources of referrals such as host hospitals, the community, and mobile vans equipped with fundus cameras [17]. Also, this screening program has leveraged smartphones to provide electronic medical reports via a messenger app.

Recently, hand-held, portable fundus cameras and smartphone-based retinal imaging have been gaining popularity as alternative to traditional tabletop retinal cameras for their cost-effectiveness and enhanced usability [18, 19]. This is important in less affluent, rural areas and developing world and in pandemic context, where qualified staff and medical equipment are lacking. Compared to conventional CFP, smartphone ophthalmoscopy has performed well in detecting DR and PDR [20]. However, as reported by the American Academy of Ophthalmology, the single-field macular centered 45° image taken with tabletop cameras has shown to be only sufficient in initial screening to recognize the presence of disease and not as a substitute for a comprehensive fundus examination [21].

Telemedicine programs based on imaging with these low-cost devices and remote interpretation have opened new avenues for assessing DR, facilitating larger population coverage and timely referral to eye specialists for those with sight-threatening DR. Also, fundus cameras with integrated AI-based algorithms can provide instant DR diagnosis, reducing the burden on healthcare systems [18].

The MII RetCam and the Remidio Fundus on Phone are two devices designed, manufactured and tested in India [22, 23] in teleophthalmology community-based DR screening, with a reported sensitivity of the latter of 100% for detecting referable DR [23].

However, future studies are warranted to test the implementation of these new devices.

Table 1 summarizes different approaches of retinal image acquisition for DR screening.

**Table 1:** Different methods of retinal image acquisition in DR teleretinal screening

**Tabletop/traditional fundus cameras** Non-mydriatic fundus camera hard-mounted in offices or mobile, on vehicles 45° and 60°	
**Ultra-wide-field cameras** [12, 13] Hard-mounted in offices or mobile, on vehicles 200° (Optos PLC, Dunfermline, Scotland, UK) 133° or 200° with montage Clarus (Carl Zeiss Meditec, Inc, Dublin, CA) 105° (Heidelberg Engineering, Inc., Heidelberg, Germany) 150° with montage or 110° in a single shot with the UWF module or 200° with three mosaic images (Eidon, ICare, Finland) 163° (Mirante, NIDEK Co., Ltd., Aichi, Japan)	
**Portable fundus cameras** [18] Portable hand-held camera (possibility for in-home testing) 40° (Visuscout 100, Carl Zeiss, Jena, Switzerland) 40° (Optomed smartscope Pro, Optomed plc, Finland) 45° × 40° (VersaCam α, Nidek Co. Ltd., Japan) 50° × 40° (Signal, Topcon, Tokyo, Japan) 50° Volk Pictor Prestige (Volk Optical, Inc, Mentor, USA) 45° Dragonfly (Eyefficient; Aurora, Ohio, USA)	
**Smartphone-based retinal imaging system** [19, 20, 22, 23] Adaptors for commercially available smartphones 25° (iExaminer adapter, WelchAllyn, Skaneateles Falls, New York)* 6–20° (D-Eye, Padova, Italy)* 20–30° (Peek Retina, Peek Vision, London) 50° (Volk iNview, Volk Optical, Inc, Mentor, USA)* 56° Paxos Scope (DigiSight Technologies; San Francisco, USA)* 45° Fundus on Phone device (Remidio Innovative Solutions, Bangalore, India)* 60° Vistaro (Remidio Innovative Solutions Pvt. Ltd., Bangalore, India) MII RetCam (Make In India Retinal Camera)	
**OCT imaging** [14, 15] Complementary strategy in DR telescreening pathway to improve accuracy of DME detection	

*OCT* optical coherence tomography, *FDA* Food & Drug Administration, *DME* diabetic macular edema

*Smartphone-based devices approved by FDA

## Clinical applications of AI for DR screening

The emphasis has been placed on AI, particularly deep learning (DL) algorithms for breakthrough performance in big data management and automated image-recognition task [24]. As opposed to other autonomous image analysis, DL algorithms substantially eliminate the manpower for hand-engineering domain-specific features and allow to explore more complex data patterns, performing an “end-to-end” learning approach. The most suitable DL architecture for image recognition is convolutional neural network (CNN), which allows computers to perform visual interpretation tasks. Computer vision and CNNs lie at the core of the interest that has sparked in image-centric specialties, like medical retina, where image-capturing devices of different modalities are used for disease assessment. The recent introduction of DL-based AI to this process has undeniable potential in staging/classifying DR on retinal images, as recognized by the international regulatory agencies [25] and is expected to improve the current disease management process.

Indeed, IDx-DR (Idx Technologies, Inc., Coralville, IA) was the first autonomous AI system cleared by the FDA in 2018 for the automated diagnosis of more-than-mild DR without clinician-assisted interpretation in the community setting [25]. The leading application of AI coupled with telemedicine is in the screening of patients in primary care and community settings. Table 2 provides a summary of DL systems with secured regulatory approval for DR detection.

**Table 2:** Summary of DL systems with the highest diagnostic performance for referable DR using fundus photographs

DL systems with secured regulatory approval for DR detection					
Study	Year	Name	Development site, Country	Regulatory status	Detection
Abramoff et al. [25]	2018	IDx-DR	University of Iowa, USA	First US FDA-approved autonomous AI device, CE-marked medical device	mtmDR
Gulshan et al. [37]	2016	Google Inc.	Google Inc., USA	CE-marked medical device	Referable/non-referable DR
Ribeiro et al. [38]	2014	RetmarkerDR	University of Coimbra, Portugal	CE-marked medical device	Disease/no-disease grading, microaneurysm turnover
Tufail et al. [39]	2015	EyeArt	Eyenuk Inc., USA	US FDA clearance, CE-marked medical device, Health Canada license	mtmDR and vtDR
Ting et al. [40]	2017	SELENA +	Singapore Eye Research Institute, Singapore	Approved by the Singapore Health Service Authority to be implemented in the national DR screening program	vtDR, glaucoma suspects and late stage AMD detection
Gonzalez-Gonzalo et al. [41]	2020	RetCAD	Thirona, The Netherlands	CE marking as a class IIa medical device in the EU	Joint detection of referable DR and AMD
Natarajan et al. [23]	2019	MEDIOS AI	Remidio, India	CE marking as a medical device in the EU	mtmDR detection

*DL* deep learning, *FDA* Food & Drug Administration, *AI* artificial intelligence, *CE* European Conformity, *mtmDR* more than mild diabetic retinopathy, *vtDR*  vision-threatening diabetic retinopathy, *DR* diabetic retinopathy, *AMD* age-related macular degeneration, *EU* European Union

Of note, Heydon et al. have demonstrated the high sensitivity of an automated retinal image analysis system to triage retinal images from the English Diabetic Eye Screening Programme, using human graders as the reference standard [26].

The rising volume of ophthalmic images for DR telescreening requires highly trained graders in a relatively cost- and resource-intensive process and is likely to outstrip workforce supply, outlining the great interest in automated image analysis. DL techniques for DR classification have shown high performance scores as compared to human reference standards, highlighting the potential for using automated DL methods as a DR screening tool [27].

The most common AI-based approaches that can be leveraged for screening purposes are the assistive semi-automated model and the stand-alone fully automated system, without any human involvement.

Thus, it has been proposed that AI could assist the ophthalmologists in reducing the workload and also non-eye health professionals in making an accurate imaging diagnosis, as well as in reading centers as an adjunct or as an alternative either to allied non-medical staff or ophthalmologists, that could assume a supervisory role [27, 28].

If, on one side, the promise of teleophthalmology is to increase the geographical coverage of clinical care for patients with DM by decentralizing services, on the other, new prospective AI applications aim to cope with the growing shortage of eye specialists by supporting the triaging of DR cases for adequate clinical care [28].

In addition, in the context of extensive lockdowns for pandemics like COVID-19, AI can help to cope with the backlog in patient care. Hence, AI implementation as part of DR telescreening model in automated classification of medical images represents an innovative, scalable and cost-effective model for DR care [29].

DL algorithms have been developed using not only standard CFP, but also other image capturing techniques. Specifically, automated segmentation algorithms can also be used on OCT volume scans, detecting intraretinal or subretinal fluid associated with DME and helping to objectively assess the treatment efficacy [30], and on UWF-CFP detecting referable DR [31] and classifying retinal vascular diseases [32].

In a screening setting, DL systems have also shown possibilities in predicting the risk of developing DR in patients with DM, with the promise to optimize screening intervals and improve vision-related outcomes [33].

Moreover, DL has yielded promising results in predicting systemic biomarkers from fundus images [34] that afford a unique opportunity for the non-invasive assessment either of DM complications or complex pathologies of ageing, including cardiovascular and neurodegenerative diseases [34, 35].

The use of AI for DR risk stratification and management, facilitating clinical therapy and predicting treatment response, could be promising, paving the way for personalized medicine. Whilst the potential benefits are undeniable, the scalability and the optimal deployment of these innovations are still unclear [36].

At the current stage, there are still limits for implementing AI in DR screening. First, ethical concerns and the need for “explainable AI” have been raised due to the lack of transparency on the features extracted by an AI model to derive its prediction, known as the “black box” problem. A potential solution is the deployment of saliency maps for the interpretability of model results [27, 31].

Second, there is no validated function to detect DME; therefore, a multi-modal method that combines fundus images and OCT scans would be ideal. Third, it remains unclear when non-DR findings, which have quantifiable retinal manifestations, should be considered referable in AI-based screening models [35].

Finally, there is a lack of reporting standards for many AI-related studies that hinders the confidence of future evaluations of this promising technology. The deployment of DL algorithms in the real-world setting is difficult and still in the early stages. Before considering any adoption in clinical practice, it should be clearly established the intended use and the degree of autonomy of the algorithms. Data integrity, protection and cyber security should be continually monitored.

## Remote monitoring

Long-term care in ophthalmology has presented a great challenge during COVID-19 pandemic because of the sheer volume of patients with chronic retinal diseases, including DR, and the limited accessibility to specialized care. A significant issue for patients with DR is the clinical burden due to the chronic and relapsing nature, which imposes frequent follow-ups for timely management.

Remote monitoring technologies have been presented as an ideal solution to provide appropriate and timely care for patients with macular diseases outside the hospital and the clinics. Specifically, investigational home OCT telemedicine systems have been developed and designed as a complement monitoring strategy for chronic retinal diseases in a primary care model. This alternative paradigm approach comprises a self-measuring OCT device, a digital platform for secure and automated data transmission, and a DL algorithm for automated quantitative OCT analysis [42]. Thus far, this novel disease monitoring approach has provided evidence for potential clinical use in neovascular age-related macular degeneration (AMD), paving the way for its application in a wide range of retinal diseases, including DME [42, 43].

Recent years have seen the widespread use of mobile devices that has fueled the rise of remote health care for monitoring patients in the home setting.

The CLEAR study has been conducted to demonstrate the reliability of the Checkup Vision Assessment System, a smart mobile-based application to measure visual acuity (VA) and Amsler grid testing in patients with AMD and DR at home. Checkup was user-friendly, and there was a good agreement between the app and in-clinic tests in the ability of monitoring patients ﻿(Pearson correlation coefficient r = 0.96) [44].

Two FDA-approved mobile medical applications for the self-assessment of vision and hyperacuity are myVisiontrack™ [45] and Alleye [46] examining a 3° and 12.7° of field, respectively.

Faes et al. showed that regular smartphone-based home monitoring could be a valuable adjunct for remote management of macular diseases, as the false alarm rate for the detection of disease progression was 6.1% (95% CI: 2.0–13.8%), the positive predictive value 80.0% (95% CI: 59.3–93.2%), and the specificity of the test was 93.8% (95% CI: 86.2–98.0%) [47].

Also, clinical outcomes of patients with DME could be improved with remote monitoring with this mobile technology. In particular, it has been demonstrated that patients able to perform mobile hyperacuity home monitoring benefit in terms of VA, and discontinue treatment less often compared to patients not using home monitoring [48].

Home monitoring could supplement telemedicine in the remote management of patients with macular diseases by reducing the cost and burden of care, as well as heightening surveillance for clinical events. The ideal remote monitoring tool should be user-friendly, at low cost, and able to automatically assess clinical changes for prompt eye specialists evaluation if necessary.

However, the safety and efficacy of remote monitoring in DR care still need to be demonstrated. Further research needs to differentiate which patients would most derive benefit from this model of care in the context of the COVID-19 pandemic and beyond [49].

## Pediatric population and telemedicine

Telemedicine can be advantageous to the management and quality of health care provided for children and adolescence with retinal diseases including DR. Telepediatrics has shown to be reliable in the diagnosis and management of pediatric ophthalmic conditions, with parents feeling comfortable with the quality of the tele-examination (98.5%) and willing to participate in another in the future (97.1%) [50]. Smartphone based-retinal imaging is particularly attractive in dealing with pediatric patients with retinal disorders, as pediatric ophthalmologists need to perform comprehensive and quick clinical examinations with adequate handling. Novel smartphone-based fundus photography devices tailored to imaging the posterior pole in pediatric population have shown to minimize children's discomfort and improve fundus photography accessibility [51, 52].

Children with DM can be easily examined with smartphone devices that are practical, easy-to-operate, lightweight with ease of image transfer. These devices may potentially increase access to diabetic eye care in areas with limited resources and closer to the households and decrease technical limitations during image acquisition in children.

Further studies are warranted to fully explore the integration of smartphone devices for pediatric diabetic telescreening. Even though there are gaps in the current knowledge, there is evidence highlighting the potential role of telemedicine in management of childhood and adolescent DR.

Recent studies have assessed the efficacy and cost-effectiveness of a non-mydriatic fundus camera with autonomous AI for the diabetic eye exam in youths, compared with a standard eye examination, showing an improved adherence to screening guidelines, that currently remain suboptimal [53, 54].

## Patient-centric digital DR healthcare framework

**Fig. 1:**
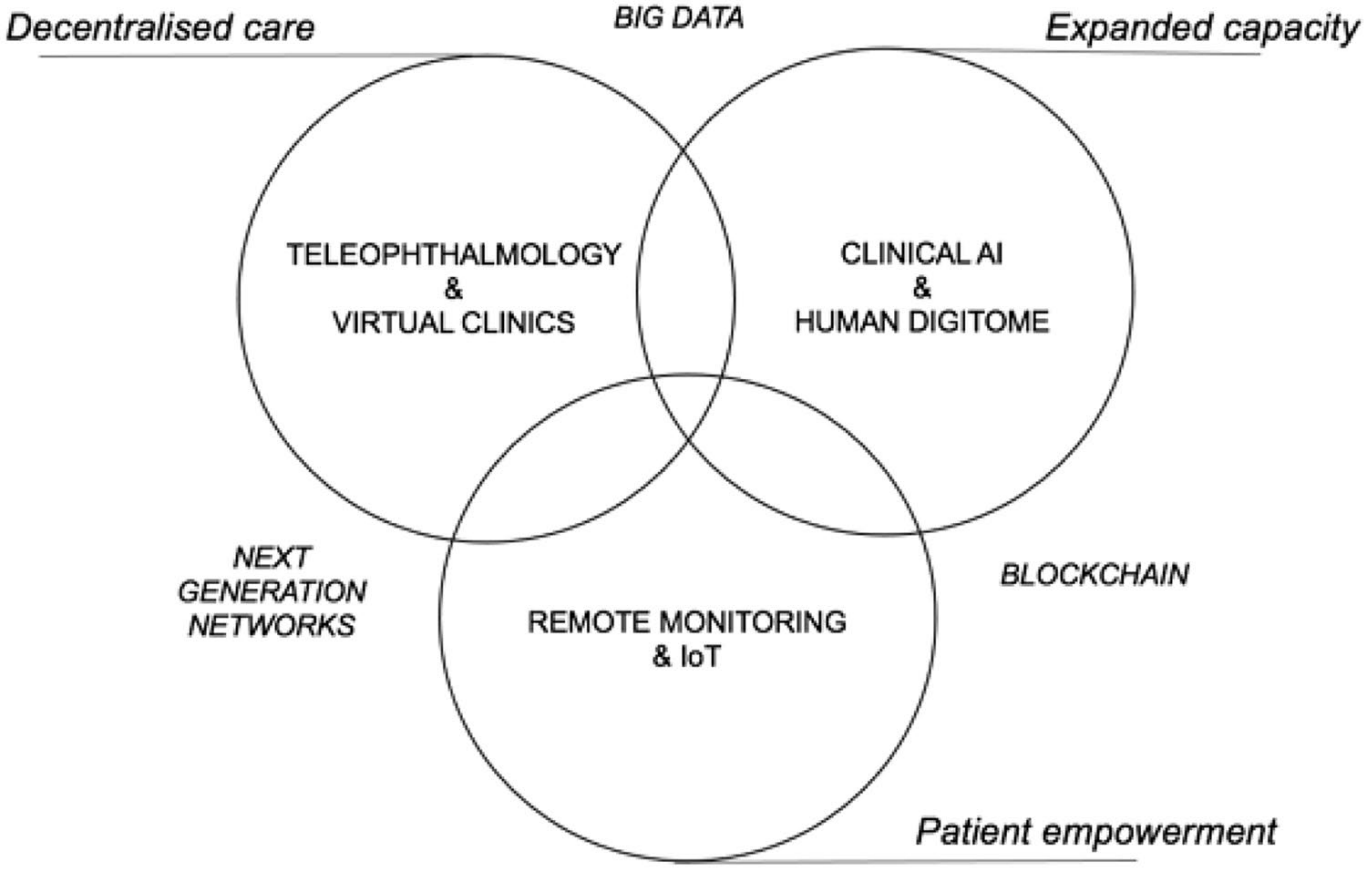
Essential building blocks for AI-assisted DR teleophthalmology screening

Figure 1 shows the core components for AI-assisted DR teleophthalmology screening that can be complementary adopted to improve health care in specific practice settings. The applications of these novel technology domains have been facilitated by COVID-19, driving a rapid expansion of telemedicine use [3, 55].

The envisioned decentralized patient-centric DR care ecosystem is conceptually organized into synergistic areas that include categories such as telemedicine, clinical AI and the Internet of Things (IoT) [56]. IoT refers to the network of interconnected of all the ordinary objects that provide independent functions through various information sensors [57]. Wearables are smart devices that can be worn or carried by individuals to monitor health outside the clinic. Recently, Internet of Wearable Things (IoWT) has emerged as a new segment of IoT [57], empowered by the tremendous growth in the number of interconnected hand-held devices from year to year [58]. Chronic disease management, such as DM, is one field of health care in which IoT is expected to have significant impacts [59]. Dwibedi et al. found that using a digital lifestyle tool at least bimonthly significantly improved metabolic control in patients with T2DM and the improvement was sustained during 2 years [59].

These new technologies can enhance healthcare quality at even lower costs, by collecting and sharing clinical data, to help patients with chronic diseases with their lifestyle management by daily monitoring of health parameters [59].

The rapid digitization of health care and the rise of these ubiquitous technologies is leading to the building of a personalized ‘human digitome’ or a ‘digital twin’, terms introduced for describing the virtual representation of a patient’s health status and disease that is constantly fed with new real-time data [60, 61]. Regarding DR care, a personalized digitome in synergy with AI could be useful for physicians to rapidly pinpoint patients with specific risk factors, support clinical decision-making in combination with traditional data, predict disease progression to create meaningful interventions and simulate treatments without risk to the subjects. It could be of great interest in providing important real-world data for digital research, therapeutics and biomarkers that are measures for explaining, influencing and predicting health-related outcomes. Data from patient history, including duration and type of DM, and other parameters such as for example blood pressure, HbA1c and visual acuity can be collected and integrated with data from other specialties, increasing the predictive and diagnostic power of the AI algorithms for DR screening.

As such, AI systems together with these emerging technologies could enhance the workflow efficiency and clinical decision making in a multidisciplinary approach for DM.

The three digital models of retinal care are integrated with and supported by three broad technology domains, including blockchain technology, big data and next-generation networks (Fig. 1) [3, 4].

Blockchain is emerging in digital health care, with a pivotal role in decentralization and cryptographic hashing to preserve hospital and patient data privacy. Research has been ongoing to deploy blockchain platforms for enabling collaborative learning techniques toward the realization of AI global model parameters [3, 62].

This decentralized digital healthcare model can facilitate the traditional public health strategies in dealing with retinal diseases such as DR, placing the patient at the center of the healthcare system. In the post-COVID-19 era, it is anticipated that this “blended” care of person and virtual care will benefit patients, if appropriately utilized.

## Challenges and future directions

Teleophthalmology screening programs have shown to be accurate and cost-effective alternatives to the traditional face-to-face examination, reaching the same clinical outcomes and high satisfaction rates among recipients and care providers. People are increasingly interested in digital health technologies, and also patients with DM are broadly in favor toward DR telescreening [63]. Keel et al. [64] have studied the feasibility and patient acceptability of AI-based DR screening, showing that 96% of the participants were satisfied and 78% preferred automated screening model over manual.

Virtual model of care for medical retina clinics, established to meet short-term needs due to COVID-19 pandemic, has shown to be an effective and safe way of triaging medical retina referrals and providing prompt treatment if necessary [65, 66]. Virtual clinic settings are meant when patients have imaging without detailed clinical encounters and discussion with an ophthalmologist at the time of imaging. This new healthcare pathway optimizes the use of current resources and workforce whilst maintaining high-quality standards of care. The implementation of multimodal UWF and OCT imaging in this form of evaluation is a promising solution to ease the burden on eye clinics [65, 66]. Therefore, it will eventually evolve into long-term solutions.

The COVID-19 pandemic has shown that the time is ripe for transforming the landscape of healthcare technology-wise; however, there needs to be hard work in this arena. Ethical, regulatory, safety and financial concerns have to be carefully considered and overcome, before the implementation of a telehealth program [67]. In addition, the issue of incomplete referral after DR telescreening is significant among individuals with sight-threatening disease, particularly in low education level and elderly population [68].

Inequalities in digital literacy lead to the exclusion of those patients that should most stand to benefit, due to health status, demographics, individual skill, access to infrastructure, training, and support.

Regardless the income setting, the most consistent barriers are functional health literacy and awareness of DR [69]. Thus, telemedicine has the potential to introduce new risks and deepen existing inequalities; educational interventions and increased public awareness are required to establish effective telemedicine DR programs [69].

WHO has recently issued guidelines for the adoption of digital technology in health care. The development of these new innovations can be exploited to tackle the common challenge among healthcare systems in achieving health coverage and sustainability in light of “benefits, harms, acceptability, feasibility, resource use and equity considerations” [70].

## Conclusions

Advances in digital health have created an ecosystem ripe for telemedicine in the field of DR to thrive, and COVID-19 pandemic has hastened this extensive operational overhaul in retinal disease management. On this basis, the recent pandemic crisis has given a significant boost to the implementation of telemedicine, highlighting its potential benefits as a complement to traditional face-to-face models of service delivery, not only in an acute care setting, but also in primary care. Healthcare system responses require a dual-track approach to boost surge capacity to contain the virus, alongside improving health outcomes for people with non-communicable diseases.

Stakeholders and policymakers should adopt a participatory approach to ensure sustained implementation of these technologies as well as physicians should keep pace with the changing models of care delivery to maximize the possible benefits and limit the potential risks of AI implementation in telemedicine services in ophthalmic care.
